# A Driving-Preference-Aware Framework for Vehicle Lane Change Prediction

**DOI:** 10.3390/s25175342

**Published:** 2025-08-28

**Authors:** Ying Lyu, Yulin Wang, Huan Liu, Xiaoyu Dong, Yifan He, Yilong Ren

**Affiliations:** 1 Key Laboratory of Advanced Vehicle Integration and Control, Changchun 130011, China; lvying@faw.com.cn (Y.L.); wangyulin11@faw.com.cn (Y.W.); liuhuan75@faw.com.cn (H.L.); dongxiaoyu5@faw.com.cn (X.D.); 2 China FAW Corporation Limited, Changchun 130011, China; 3 College of Communication Engineering, Jilin University, Changchun 130012, China; 4 School of Transportation Science and Engineering, Beihang University, Beijing 102206, China; yiifanhe@buaa.edu.cn

**Keywords:** lane change prediction, driving preference, contrastive learning, feature fusion

## Abstract

With the development of intelligent connected vehicle and artificial intelligence technologies, mixed traffic scenarios where autonomous and human-driven vehicles coexist are becoming increasingly common. Autonomous vehicles need to accurately predict the lane change behavior of preceding vehicles to ensure safety. However, lane change behavior of human-driven vehicles is influenced by both environmental factors and driver preferences, which increases its uncertainty and makes prediction more difficult. To address this challenge, this paper focuses on the mining of driving preferences and the prediction of lane change behavior. We clarify the definition of driving preference and its relationship with driving style and construct a representation of driving operations based on vehicle dynamics parameters and statistical features. A preference feature extractor based on the SimCLR contrastive learning framework is designed to capture high-dimensional driving preference features through unsupervised learning, effectively distinguishing between aggressive, normal, and conservative driving styles. Furthermore, a dual-branch lane change prediction model is proposed, which fuses explicit temporal features of vehicle states with implicit driving preference features, enabling efficient integration of multi-source information. Experimental results on the HighD dataset show that the proposed model significantly outperforms traditional models such as Transformer and LSTM in lane change prediction accuracy, providing technical support for improving the safety and human-likeness of autonomous driving decision-making.

## 1. Introduction

Frequent road traffic accidents have become a serious social problem [1]. This stems from factors like poor safety awareness of participants, traffic rule violations, inadequate road facilities, and management loopholes, leading to heavy casualties and huge property losses [2]. The causes of traffic accidents are complex, involving human factors, road design, vehicle performance, environmental conditions, and traffic management [3]. Among these, human factors are particularly prominent—statistics show that approximately 94% of traffic accidents are caused by driver errors [4].

Lane changing is one of the most common maneuvers in daily driving and plays a critical role in maintaining road safety and traffic flow. When executing a lane change, drivers must accurately judge the timing by considering dynamic factors such as the instantaneous speed and inter-vehicle distance of surrounding vehicles. However, due to the inherent limitations of human cognitive capacity and information processing ability, drivers often fail to fully integrate real-time information about road conditions, the behavior of nearby vehicles, and the movement of pedestrians. This can lead to misjudgment of potential risks and increase the likelihood of side collisions or rear-end accidents. Studies have shown that approximately 27% of traffic accidents are related to improper lane changes [5], underscoring the importance of accurate lane change behavior prediction.

Autonomous driving technology is widely regarded as a key solution to mitigate these issues [6]. Equipped with advanced sensors such as LiDAR and cameras, autonomous vehicles can perceive their surroundings with high precision. By leveraging machine learning and deep learning algorithms, they can learn complex driving patterns and respond quickly to changing traffic environments to avoid risks. However, current road traffic systems remain in a transitional phase where autonomous and human-driven vehicles coexist. In such mixed traffic scenarios, autonomous vehicles must frequently interact with human drivers, whose behavior introduces significant uncertainty due to individual driving preferences. Differences in driving habits among individuals can lead to distinct decision-making strategies, and even the same driver may exhibit varied behaviors under different environmental conditions [7]. This behavioral uncertainty makes lane change prediction—which already involves complex multi-vehicle, multi-lane interactions—even more challenging, thereby impacting both traffic efficiency and decision-making safety in mixed autonomy environments.

In light of this, this study adopts a data-driven approach using real-world vehicle trajectory data, with a focus on modeling driving preference—a key latent feature influencing behavior that has been insufficiently addressed in existing research. While deep learning-based methods for capturing driving styles have been widely reported, most rely on supervised learning with manually annotated labels, which introduces human bias and limits scalability. In contrast, this work constructs a structured driving operational picture (DOP) by encoding vehicle dynamics and statistical features, offering a more comprehensive representation of driving behaviors compared to conventional handcrafted features.

Building upon the SimCLR framework, we propose an unsupervised contrastive learning-based preference feature extractor—distinguishing it from existing SOTA methods that often depend on labeled data—to capture high-dimensional semantic features of driving preferences. This approach not only avoids human bias inherent in traditional methods but also learns more nuanced representations that effectively differentiate aggressive, normal, and conservative driving styles.

On this basis, we develop a dual-branch prediction model that fuses explicit features (vehicle states and environmental parameters) with implicit driving preferences—an integration strategy rarely explored in existing lane change prediction models. One branch, based on an enhanced Transformer architecture, models sequential features, while the other integrates pre-trained preference representations. The feature fusion module enables efficient integration of multi-source information, addressing the limitation of existing models that either underutilize temporal features or oversimplify preference representation.

This research aims to provide a fine-grained understanding of lane change behavior and enable accurate recognition and prediction, thereby offering theoretical support for behavioral traffic studies and promoting the development of personalized driver-assistance systems and human-like decision-making in autonomous driving.

## 2. Related Work

Deep vehicle lane change behavior prediction is a core research topic in intelligent transportation systems and autonomous driving, aiming to improve traffic safety and efficiency by modeling driver decision-making mechanisms. Existing studies can be broadly categorized into rule-based models and learning-based models, which explore the underlying logic of lane change behavior from the perspectives of physical laws and data-driven learning, respectively. The following sections review key developments in both areas.

### 2.1. Rule-Based Lane Change Prediction Models

Rule-based models describe lane change decisions through predefined physical laws, logical rules, or optimization objectives, abstracting driver behavior into quantifiable constraints. These models are highly interpretable and were widely adopted in early traffic simulation and driver assistance systems.

#### 2.1.1. Logical Rule and Optimization Models

The Gipps model [8] was the first systematic rule-based model for lane changing, incorporating traffic signals, obstacles, and other environmental factors to formulate decision logic in urban scenarios. This model laid the foundation for subsequent studies. Building on this framework, Yang et al. [9] introduced the classifications of Mandatory Lane Changes (MLC) and Discretionary Lane Changes (DLC). MLC refers to lane changes required by road conditions, while DLC involves driver-initiated maneuvers to improve efficiency—this classification remains a fundamental paradigm in lane change research. Zhang et al. [10] further categorized the Gipps model under MLC and proposed the Multi-Region Simulation (MRS) model, enhancing simulation realism by refining car-following and lane change logic across different scenarios.

Hidas [11,12] proposed the SITRAS model, which introduced the concept of cooperative lane changing, emphasizing dynamic coordination between the lane-changing vehicle and vehicles in the target lane—specifically, the inter-vehicle distance first increases and then decreases during the lane change. This mechanism enables realistic simulation of traffic flow interactions under congestion, and experiments have demonstrated its effectiveness in reproducing traffic speed–flow relationships in such conditions. The MOBIL model by Kesting et al. [13] optimizes lane change decisions by minimizing overall braking in the system. It balances personal and system-level objectives through three constraints: incentive (improving self-efficiency), safety (avoiding collisions), and cooperation (promoting traffic flow).

#### 2.1.2. Cellular Automata and Fuzzy Logic Models

Cellular Automata (CA) divide roads into discrete cells and simulate vehicle movements using simple rules, making them widely used in traffic flow simulations. The NaSch model proposed by Nagel and Schreckenberg [14] was the first CA-based traffic model, simulating single-lane traffic flow through rules like acceleration, deceleration, and random slowing. Rickert et al. [15] extended it to multi-lane scenarios with symmetric and asymmetric lane change rules. Wang et al. [16] introduced “squeezing lane change” rules to model vehicle interactions under congested conditions. Chinese scholars further optimized CA models for specific scenarios such as ramp entrances and upstream of intersections [17], enhancing applicability by adjusting cell transition probabilities.

Fuzzy logic models use membership functions to handle uncertainty in driving decisions, aligning more closely with human subjective judgment. Shi and Zhang [18] developed a fuzzy control model that integrates driver experience and safety thresholds, outputting lane change probabilities through fuzzy inference, thus reflecting individual driver differences. Balal et al. [19] validated the effectiveness of fuzzy inference for discretionary lane change decisions using the NGSIM dataset.

#### 2.1.3. Game Theory and Utility-Based Models

Game theory treats lane change behavior as a multi-agent decision-making process and is suitable for modeling strategic interactions among vehicles. Kita [20] applied non-zero-sum game theory to analyze merging scenarios at highway ramps, revealing equilibrium strategies in speed adjustments between merging and through vehicles. Kim and Langari [21] employed mixed-motive game theory in autonomous vehicles by combining deterministic and probabilistic strategies to optimize decision rewards. Experimental results showed that this approach improved the overall utility of all participating vehicles.

Utility theory quantifies lane change decisions from a “cost–benefit” perspective. Ahmed et al. [22] proposed a four-level decision-making framework that decomposes the lane change process into three stages: considering lane change, choosing the target lane, and accepting a gap. By integrating time cost, safety risk, and other factors into a utility function, the model provides a quantitative tool for multi-objective decision-making.

### 2.2. Learning-Based Lane Change Prediction Models

With advancements in sensing technologies and machine learning, data-driven approaches have become mainstream. These models learn behavior patterns from large-scale trajectory data and do not rely on handcrafted rules, making them better suited for complex and dynamic traffic scenarios.

#### 2.2.1. Traditional Machine Learning Methods

Support Vector Machines (SVMs) and Random Forests were widely used in early lane change prediction tasks. Mandalia et al. [23] used SVMs to classify vehicle environmental and behavioral features to infer lane change intentions. Vallon et al. [24] further employed SVMs to capture driver preference patterns, enabling the model to predict personalized lane changes even without turn signal activation. Ensemble learning methods such as Random Forests have also shown advantages. Gu et al. [25] proposed an ensemble learning model that addressed the limitations of rule-based models in capturing the entire lane change process, improving prediction robustness through a voting mechanism across multiple decision trees.

Hidden Markov Models (HMMs) and their variants have been effective in modeling sequential behaviors. Zheng et al. [26] combined vehicle dynamics inputs with HMMs to predict lane change decisions across multiple lanes based on hidden state sequences. Zhao et al. [27] introduced the LCCF model, which integrates Gaussian Mixture HMMs to provide early warnings of lane changes 0.6–1.3 s in advance, giving autonomous vehicles sufficient time for reaction. Bayesian approaches have demonstrated advantages in handling uncertainty. Song et al. [28] calibrated driver models using Bayesian networks to output probabilistic distributions of lane change behaviors, enhancing risk awareness in decision-making.

#### 2.2.2. Deep Learning Methods

Deep learning models leverage multi-layer neural networks to automatically extract high-level features, offering significant advantages in handling high-dimensional and nonlinear traffic data. Huang et al. [29] demonstrated the potential of Deep Neural Networks (DNNs) in lane change prediction. Their model took historical trajectories and surrounding distances as inputs and achieved better fitting performance than traditional methods. Mo et al. [30] proposed a hybrid CNN-LSTM model, which is representative in temporal modeling. The CNN layer extracted spatial interaction features between ego and surrounding vehicles, while the LSTM layer captured temporal dependencies, enabling lane change predictions with an average lead time of 3.6 s—providing ample response time for decision-making.

The introduction of autoencoders and attention mechanisms further improved feature learning efficiency. Gu et al. [31] used Deep Autoencoders (DAEs) to compress sequential driving information and combined them with a Bayesian-optimized XGBoost model to retain key patterns while reducing feature dimensionality. For edge cases such as failed lane changes, Xing et al. [32] proposed an event detection method based on the Mexican hat wavelet and applied a GA-XGBoost model to predict failed lane changes, offering new tools for risk avoidance.

In summary, existing studies have not fully addressed three core issues. Firstly, mainstream models such as LSTM and Transformer mostly focus on modeling explicit temporal features, with insufficient exploration of implicit factors (like driving preferences) that influence lane change decisions. Secondly, existing research related to driving preferences mostly relies on supervised learning, requiring manual annotation of driving style labels, which easily introduces subjective biases and limits generalization. Thirdly, at the feature fusion level, there is a lack of efficient integration mechanisms for explicit temporal features (e.g., vehicle dynamics) and implicit preference features.

To this end, this study proposes targeted solutions. First is constructing an unsupervised contrastive learning model based on the SimCLR framework to automatically extract high-dimensional driving preference features from driving operational pictures (DOPs) without manual annotation, overcoming the limitation of LSTM and Transformer that rely on supervised learning. Second is designing a dual-branch prediction architecture—one branch models explicit temporal features based on an enhanced Transformer, and the other integrates pre-trained implicit preference features. Through feature-level fusion, complementary multi-source information is achieved, retaining Transformer’s ability to capture long-term temporal dependencies while compensating for its deficiency in modeling implicit preferences. Finally, by combining unsupervised learning with dual-branch fusion, the shortcomings of existing models in implicit feature mining and multi-source information integration are addressed, providing a more comprehensive and robust technical path for lane change behavior prediction.

## 3. Methodology

### 3.1. Construction of the Driving Operational Picture

Traditional methods for representing driving preferences can be broadly categorized into two approaches: subjective questionnaire-based assessments and objective data-driven mining. Subjective assessments typically include self-evaluations by drivers and expert scoring systems [33]. In contrast, objective data mining formulates the representation of driving preferences as a classification problem, wherein driving preferences are described using specific features (e.g., vehicle kinematics), and classification algorithms are applied to learn these patterns.

Although the data-driven approach improves upon subjective methods by using objective feature information, it still fundamentally relies on supervised learning, which requires manually annotated class labels. This dependency can introduce significant human error, especially when handling large-scale datasets. Moreover, by casting driving preference modeling as a discrete classification task, this approach assumes uniformity among preferences within each category. While instances in the same class may share similar low-dimensional outputs, they can exhibit substantial semantic variation in high-dimensional feature space, leading to additional inaccuracies.

Contrastive learning offers two key advantages that address these shortcomings. First, it eliminates the need for manually labeled data. Second, it learns high-dimensional feature representations, which better capture the nuanced semantics of driving behavior. Therefore, this study adopts a contrastive learning approach based on the SimCLR framework to extract driving preference features in an unsupervised manner.

Originally proposed for computer vision tasks, SimCLR requires carefully designed input representations when applied to non-image domains such as driving behavior. Thus, a key challenge lies in how to effectively encode driving preference information into a format suitable for the model.

To this end, we introduce the driving operational picture (DOP) as the input representation of driving preference features. The effectiveness of this representation has been validated in prior studies by Zhang et al. [34] and Li et al. [35].

**Figure 1:**
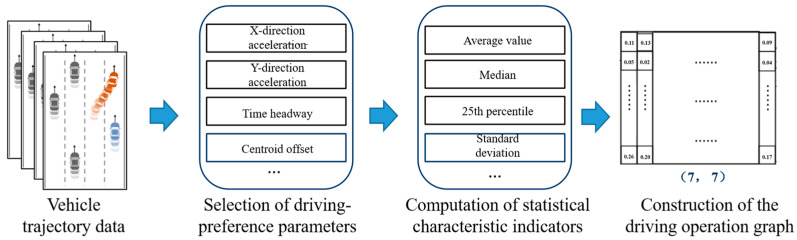
Construction process of the driving operational picture (DOP).

As illustrated in Figure 1, the construction process of the DOP begins by extracting key features from vehicle trajectory data that reflect individual driving preferences. A total of seven features are selected: Lateral velocity (xVelocity), Lateral acceleration (xAcceleration), Longitudinal velocity (yVelocity), Longitudinal acceleration (yAcceleration), Lateral displacement from lane center (delta_y), Time headway (thw), and Distance headway (dhw). All features are normalized to ensure consistency and comparability across different drivers and driving conditions:

(1) $$x^{\prime }=\frac{x-x_{min}}{x_{max}-x_{min}}\;\;$$

Here, x represents the raw feature value, while $x_{max}$ and $x_{min}$ denote the maximum and minimum values of that feature across the entire dataset, respectively.

For each of the seven selected features, we compute the following seven statistical descriptors over the duration of the selected trajectory: mean, maximum, minimum, median, 25th percentile, 75th percentile, and standard deviation.

This results in a dense driving preference feature matrix, where each row corresponds to one vehicle feature and each column corresponds to one statistical function. The final driving operational picture (DOP) is a 7 × 7 matrix, with each vehicle instance associated with a unique DOP.

### 3.2. Driving Preference Feature Extractor

#### 3.2.1. Feature Extractor Training Network

**Figure 2:**
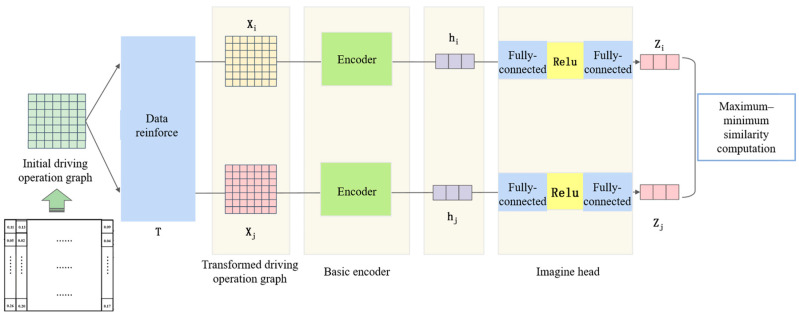
Training network of the feature extractor.

As shown in Figure 2, the training network of the feature extractor consists of three main components: a data augmentation module, an encoder module, and a projection head.

The constructed raw DOP is first passed through the data augmentation module to generate a pair of augmented samples. These samples are then fed into the encoder to obtain high-dimensional feature representations. The encoded features are subsequently mapped to a lower-dimensional space via the projection head. Finally, similarity-based contrastive learning is applied to encourage the model to learn discriminative feature representations that distinguish between different inputs.

#### 3.2.2. Driving Operational Picture Feature Augmentation

The feature augmentation process consists of two main parts: preprocessing and image augmentation.

(1) DOP Preprocessing

The preprocessing of the DOP involves two main steps: channel dimension expansion and size enlargement. First, channel dimension expansion converts the 2D array into a 3D tensor. This is necessary because in PyTorch 2.5.1, image data is typically represented in the format (C, H, W). Since the DOP is treated as a pseudo-image, an additional channel dimension is added to create a single-channel DOP tensor. Next, the size of the DOP is enlarged. On the one hand, this ensures compatibility with the input size requirements of the encoder; on the other hand, it introduces greater diversity into the dataset. Enlarging the DOP helps the model learn more robust features, mitigates training instability, and ultimately improves both training effectiveness and model performance. Additionally, image augmentation methods operate within a certain size range—by enlarging the DOP, more transformation space is created for data augmentation, leading to improved augmentation quality.

(2) Image Augmentation

Random Resized Crop: Random resized cropping is a data augmentation technique that randomly crops regions of an image to simulate different views, thereby increasing data diversity and improving the generalization ability of the model. By focusing on different regions, the model learns to be robust to local variations and avoids overfitting. The operation is defined by Equation (2):

(2) $$new\_img=img\left[x_{1}:x_{1}+w,\;y_{1}:y_{1}+h\right]$$

where $\left(x_{1},y_{1}\right)$ is the randomly selected starting point, satisfying $0\leq x_{1}\leq W-w$ and $0\leq y_{1}\leq H-h$. $\left(W,H\right)$ and $\left(w,h\right)$ are the dimensions before and after cropping, respectively.

Gaussian Blur: Gaussian blur applies a smoothing effect to the image, blurring fine details so the model can focus on global structural features rather than noisy local information. The operation convolves the image with a Gaussian kernel, as defined in Equation (3):

(3) blurred_img=img∗G

where ∗ denotes convolution, and G is the Gaussian kernel computed as:

(4) $$G(x,y)=\frac{1}{2\pi \sigma^{2}}e^{-\frac{x^{2}+y^{2}}{2\sigma^{2}}}$$

Here, (x,y) denotes the kernel coordinates, and σ is the standard deviation controlling the degree of blurring. In this study, σ=0.1.

Random Horizontal Flip: Random horizontal flipping reverses the image along the vertical axis, helping the model learn symmetry and become more robust to directional changes. By introducing randomness, this operation prevents the model from overfitting to directional biases in the training data. The flipping operation is defined as:

(5) $$lipped_{img}=\left\{\begin{array}{c} img\;\;\;\;\;\;\;\;\;\;\;\;\;\;\;\;\;\;\;with\;probability\;1-p\\ img\left[:,∷-1,:\right]\;\;\;\;\;\;\;with\;probability\;p \end{array}\right.$$

Here, ∷−1 represents reversed indexing along the width dimension, achieving the horizontal flip.

**Figure 3:**
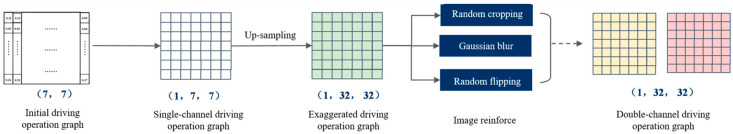
Illustration of data augmentation for driving operational pictures.

After applying the above augmentation operations, we obtain a pair of DOP views that serve as positive samples in contrastive learning. An illustration of this process is shown in Figure 3.

#### 3.2.3. Feature Extractor and Projection Head

**Figure 4:**
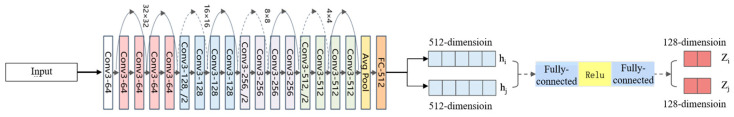
Illustration of feature extraction and projection head.

The generated pairs of DOP are used as the input to the feature extractor. Each pair is processed sequentially through feature extraction and projection, after which similarity computation is performed to guide the contrastive learning process. A schematic illustration of this process is shown in Figure 4.

ResNet-18 is selected as the encoder for feature extraction in this study. Originally proposed by Kaiming He et al. [36] at Microsoft Research in 2015, ResNet-18 is a deep convolutional residual neural network that has been widely applied in computer vision tasks. The architecture consists of 17 convolutional layers and 1 fully connected layer (excluding auxiliary layers such as pooling and batch normalization). The overall structure is organized into several modules, including an initial convolution-pooling block and four residual blocks (layer1, layer2, layer3, and layer4), each containing multiple basic residual blocks.

The choice of ResNet-18 over deeper variants such as ResNet-34 or ResNet-50 is motivated by two main considerations:

Task Requirements: For small- to medium-scale datasets, ResNet-18 is sufficiently capable of extracting meaningful features and learning effective image representations. Deeper networks like ResNet-50 have a much larger number of parameters, which can lead to overfitting, especially during contrastive learning, where generalization is critical.

Computational Efficiency: Compared to ResNet-34 or ResNet-50, ResNet-18 has significantly fewer layers and parameters—only about one-quarter the number of parameters of ResNet-50. Contrastive learning also requires relatively large batch sizes to provide sufficient negative samples, which further increases computational cost. Thus, ResNet-18 offers a good trade-off between performance and efficiency.

After passing through ResNet-18, each DOP is encoded into a 512-dimensional feature vector. This vector is then passed to a projection head for further transformation. The projection head is implemented as a two-layer multilayer perceptron (MLP): The first fully connected layer maintains the same input and output dimensionality to facilitate better feature separation and aggregation. A ReLU activation function is applied to introduce non-linearity, enriching the feature representations. Finally, the second fully connected layer maps the features into a space suitable for contrastive learning objectives.

#### 3.2.4. Loss Function and Optimizer

**Figure 5:**
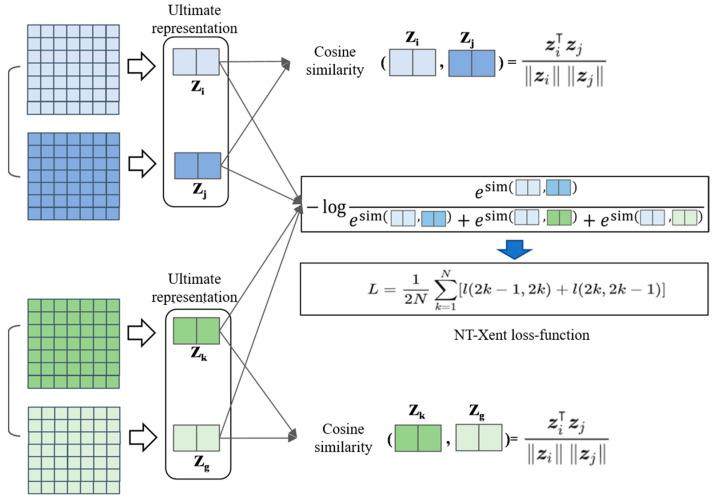
Illustration of the loss function computation.

In this study, we adopt the Normalized Temperature-scaled Cross Entropy Loss (NT-Xent Loss) as the objective function for contrastive learning. A schematic diagram illustrating this loss computation is shown in Figure 5.

First, the definitions of positive and negative sample pairs are established. Positive pairs refer to different augmented views derived from the same original image. For example, in Figure 5, the light blue and dark blue DOPs form a positive pair, as do the light green and dark green DOPs. In contrast, negative pairs consist of augmented views from different original images, such as the light green DOP paired with either the light blue or dark blue DOPs.

The objective of the contrastive loss is to maximize the similarity between positive pairs, encouraging augmented versions of the same image to be close in the feature space, while simultaneously minimizing the similarity between negative pairs, pushing different images’ augmented versions far apart in the feature space. This is achieved by calculating the similarity between samples using cosine similarity, combined with a cross-entropy loss formulation for optimization. The loss function is defined as follows:

(6) $$l_{i,j}=-log\frac{\exp\left(sim\left(z_{i},z_{j}\right)/\tau \right)}{\sum_{k=1}^{2N}\;1_{\left[k\neq i\right]}exp\left(sim\left(z_{i},z_{k}\right)/\tau \right)}$$

where $l_{i,j}$ denotes the contrastive loss for the positive pair $\left(i,j\right)$, and τ is the temperature parameter that adjusts the sensitivity of similarity scores between samples. The summation $\sum_{k=1}^{2N}\;$ is taken over all other samples, where 2N represents the total number of samples in the batch (each original sample generates two augmented views, resulting in 2N samples in total). The indicator function $1_{\left[k\neq i\right]}$ equals 1 if k≠i, ensuring that the similarity of a sample with itself is excluded from the negative samples.

The similarity function $sim\left(z_{i},z_{j}\right)$ measures the similarity between two feature vectors $z_{i}$ and $z_{j}$ using cosine similarity, defined as:

(7) $$sim\left(z_{i},z_{k}\right)=cos(\theta )=\frac{z_{i}\cdot z_{j}}{\left\|z_{i}\|\|z_{j}\right\|}$$

Here, $z_{i}$ and $z_{j}$ represent the feature vectors output by the projection head.

For all samples in a given batch, the NT-Xent Loss computes the contrastive loss for each positive pair, and the final loss is obtained by averaging over all positive pairs:

(8) $$L=\frac{1}{2N}\sum_{k=1}^{N}\left(l_{2k-1,2k}+l_{2k,2k-1}\right)$$

Here, k denotes the index of the original image in the batch, and 2k−1 and 2k represent the two augmented views generated from that image. The total loss function includes bidirectional computation for each positive pair to ensure symmetrical learning.

For optimization, we continue to use the Adam optimizer, consistent with previous configurations.

### 3.3. Dual-Branch Lane Change Behavior Prediction Model

#### Model Architecture

**Figure 6:**
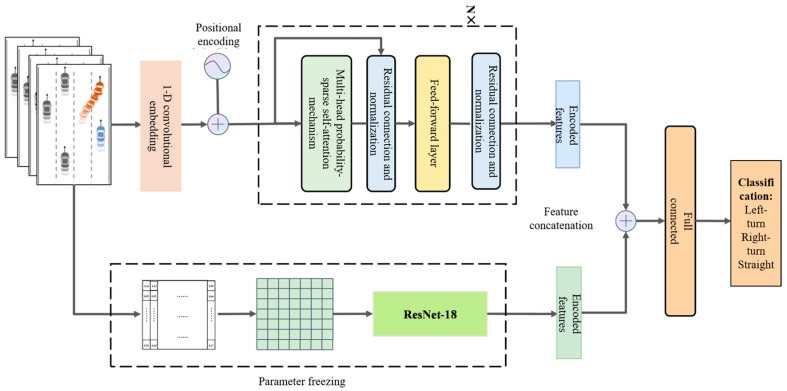
Overall architecture of the proposed model.

To make effective use of features and improve prediction accuracy by integrating multi-source information (i.e., sequential behavioral features and driver preference features), we propose a feature-level fusion model that combines the driver preference feature extractor with a lane change behavior prediction model based on long-term temporal dependency modeling. The fusion is performed via feature concatenation, where implicit driver preference features are combined with explicit behavioral features. As illustrated in Figure 6, the model consists of two branches: One branch processes explicit features, producing a high-dimensional representation using a probabilistic sparse encoder. The other branch encodes implicit features via the ResNet-18-based driver preference extractor, generating a semantic feature representation. The outputs of both branches are concatenated, followed by a fully connected layer. After a linear transformation and non-linear activation, the final prediction is generated by a Softmax layer, which outputs the most probable classification result.

During training, the parameters of the ResNet-18 branch are frozen, and only the parameters of the other branch and the fully connected layers are updated via backpropagation.

## 4. Experiments

### 4.1. Introduction of the Dataset

Our study uses the HighD dataset. The HighD dataset is a large-scale collection of naturalistic vehicle trajectories recorded by drone-borne high-resolution cameras over six German highway segments. It contains 16.5 h of video, yielding 110,500 vehicles and 45,000 km of travel, with 5600 complete lane changes automatically extracted by computer-vision algorithms whose positioning error is typically < 10 cm. Each vehicle’s type, dimensions, and maneuvers are provided at 25 Hz, enabling centimeter-level trajectory reconstruction. Originally designed for safety validation of highly automated driving systems, HighD is also widely used for traffic pattern analysis, driver model parameterization, and microscopic traffic simulation.

Before utilizing the HighD dataset, it is essential to take into account the influence of traffic flow conditions, vehicle types, road characteristics, weather factors, and driver heterogeneity on lane-changing behavior. Therefore, data preprocessing and feature extraction are required prior to model development.

(1) Vehicle category classification: The numLaneChanges field in the tracksMeta.csv file is traversed to classify vehicles into lane-changing and lane-keeping categories, and their corresponding vehicle IDs are stored separately. Based on these vehicle IDs, the associated trajectory data from the tracks.csv file are retrieved to construct the lane-changing vehicle subset and the lane-keeping vehicle subset, respectively.

(2) Lane information extraction: Using the upperLaneMarkings and lowerLaneMarkings fields from the recordingMeta.csv file, the number of lanes at each recording location is determined. Each lane is then labeled accordingly for different road configurations, including four-lane, six-lane, and seven-lane bidirectional roads.

(3) Feature extraction for lane change prediction: Lane change prediction features are categorized into three aspects: ego vehicle state, surrounding vehicle state, and roadway conditions. Specifically, the ego vehicle state features include xVelocity, yVelocity, xAcceleration, and yAcceleration from the tracks.csv file, as well as the class field from the tracksMeta.csv file. Roadway condition features include left_lane_exist and right_lane_exist, derived from lane information in the recordingMeta.csv file as obtained in step (2).

(4) Surrounding vehicle state computation: Eight surrounding vehicles are considered, including those in the front of the same lane, front of the left lane, front of the right lane, parallel in the left lane, parallel in the right lane, behind in the same lane, behind in the left lane, and behind in the right lane. By combining information from the *tracksMeta.csv* and *tracks.csv* files, the Time-to-Collision (TTC) between the ego vehicle and each surrounding vehicle is computed to characterize their interactions.

(5) Critical point identification and lane change direction determination: Since the initiation time of lane-changing behavior varies across vehicles, the conventional approach of defining the lane change process as a fixed time window before and after the lane change event introduces significant errors and is insufficient for early prediction. Therefore, it is necessary to identify the critical onset point of lane changing and to determine its direction accordingly. This paper defines a lane change initiation discriminant, denoted as $D_{diff}$:

(9) $$D_{diff}=\frac{∆y}{height}\geq \frac{1}{2}$$

where ∆y denotes the lateral deviation between the vehicle’s centerline in the y direction and the centerline of its current lane, while *height* represents the vehicle width. A lane change maneuver is considered to be initiated once the longitudinal distance (in the x direction) between the vehicle’s centerline in the y direction and the lane centerline exceeds half of the vehicle width.

Also, we divide the lane-changing behavior into the decision-making phase (generating the intention to change lanes), the reaction phase (preparing for lane-changing actions, such as turning on the turn signal), and the execution phase (the vehicle crossing the lane line). After determining the critical point of vehicle lane change, it is necessary to intercept the lane change time window forward by time steps as the input data for vehicle lane change behavior prediction. The selection of the lane change time window has a significant impact on the prediction results. If the time window is too short, it cannot cover the complete lane change behavior cycle; if the time window is too long, it will dilute the importance of key features. Generally speaking, 2–3 s is an empirical value in most studies. For example, Waymo’s prediction module often uses a 2.5 s window. Consistent with previous studies, this paper adopts a 2 s time window (i.e., 50 frames of data) for the HighD dataset. The 2 s window corresponds to the early stage of lane-changing decision-making: that is, the period from when the vehicle begins to generate the intention to change lanes, prepares to execute lane-changing actions (such as adjusting vehicle speed, observing surrounding vehicles), to just before it is about to cross the lane line.

Finally, the input feature information for vehicle lane change behavior prediction covering the surrounding vehicle states, self-vehicle states, and road conditions is obtained. The input data format is (17590, 50, 16), which means there are a total of 17,590 vehicles, each with 50 frames of input data, and the feature dimension is 16.

### 4.2. Analysis of Feature Extractor Results

In this study, we adopt two commonly used evaluation metrics in computer vision classification tasks: Top-1 Accuracy (top1acc) and Top-5 Accuracy (top5acc).

Top-1 Accuracy refers to the proportion of input samples for which the model’s most probable prediction (i.e., the class with the highest predicted probability) matches the ground truth label. In other words, it measures how often the model’s top prediction is correct. The formula is defined as follows:

(10) $$Top-1\;Accuracy=\frac{Number\;of\;correct\;top-1\;predictions}{Total\;number\;ofsamples}$$

Top-1 Accuracy reflects whether the model’s most confident prediction for an input image is correct, making it a highly intuitive performance evaluation metric. A high Top-1 Accuracy indicates that the model is capable of effectively mapping image features to the correct class in the label space, thereby accurately identifying the target category.

Top-5 Accuracy is defined as the proportion of input samples for which at least one of the top five predicted classes matches the ground truth label. This metric takes into account multiple possible predictions made by the model, rather than relying solely on the most probable one. The formula is given as follows:

(11) $$Top-5\;Accuracy=\frac{Number\;of\;correct\;top-5\;predictions}{Total\;number\;of\;samples}$$

Compared to Top-1 Accuracy, Top-5 Accuracy is a more relaxed evaluation metric. It is particularly suitable for assessing model performance in multi-class classification tasks, where the differences between certain classes can be very subtle. This is especially relevant when features are learned from unlabeled datasets via contrastive learning and later evaluated in supervised settings. In such cases, Top-5 Accuracy offers a more comprehensive performance perspective.

**Figure 7:**
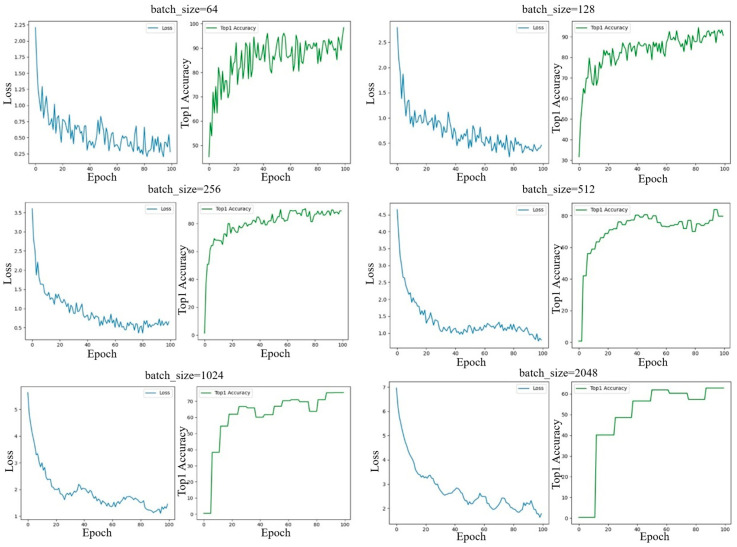
Experimental results with different batch sizes.

The batch size plays a critical role in training the proposed model, as it directly affects the number of positive and negative sample pairs in contrastive learning, the effectiveness of the loss function, and ultimately the overall model performance. To explore the optimal configuration, we experimented with six different batch sizes: 64, 128, 256, 512, 1024, and 2056. The corresponding experimental results are illustrated in Figure 7.

**Figure 8:**
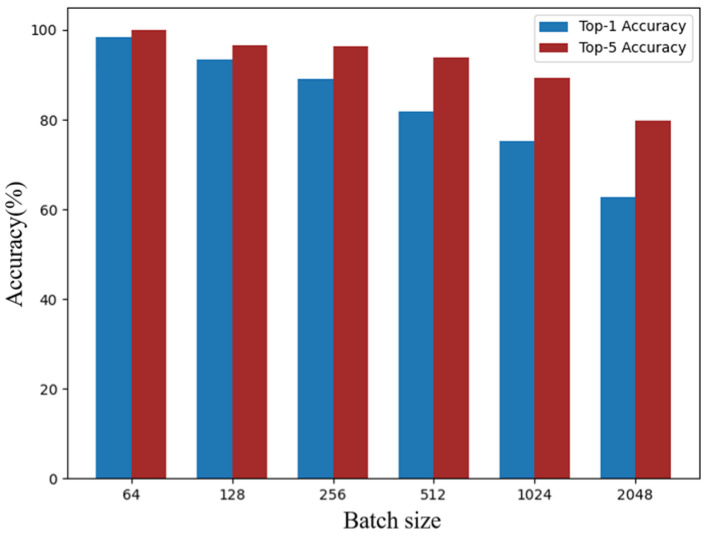
Accuracy comparison across different batch sizes.

The final results of Top-1 Accuracy and Top-5 Accuracy under different parameter settings are compared in Figure 8.

An analysis of Figure 8 reveals an abnormal step-like pattern in the curves of loss and Top-1 Accuracy when the batch size becomes relatively large (i.e., batch_size ≥ 512) under a fixed learning rate of 0.0003. Specifically, for certain batch sizes, the metric values remain constant without noticeable changes. Generally, contrastive learning tends to perform better with larger batch sizes, as more negative samples allow the loss function to more effectively optimize the feature space and learn more discriminative feature representations. However, in our case, several factors limit the benefits of using large batch sizes.

Dataset scale: Compared to conventional image classification datasets, the dataset in this study is relatively small. The driving operational pictures (DOPs) are constructed from vehicle trajectories, with only about 10,000 samples, whereas datasets like ImageNet contain over one million labeled images.

Image size: Each DOP has a resolution of 32 × 32, which is much smaller than typical image inputs in computer vision tasks.

Feature diversity: Prior studies on driver preferences usually categorize drivers into three types. While this classification is subjective and coarse, it indirectly implies that within each category, the high-dimensional feature variation may be limited. This suggests that the diversity of high-dimensional features in DOPs is lower than that of natural images, making extremely large batch sizes less suitable for this task.

From Figure 8, it is evident that both Top-1 and Top-5 Accuracy gradually decrease as the batch size increases, especially when batch_size ≥ 512. With smaller batch sizes, the model achieves higher accuracy. Although accuracy slightly decreases with increasing batch size, it is important to note that the number of candidate negative samples also increases proportionally. Notably, at batch_size = 256, the Top-5 Accuracy remains relatively stable, suggesting a favorable balance. Based on this analysis, we select batch_size = 256 as the optimal setting for the driver preference feature extractor.

**Figure 9:**
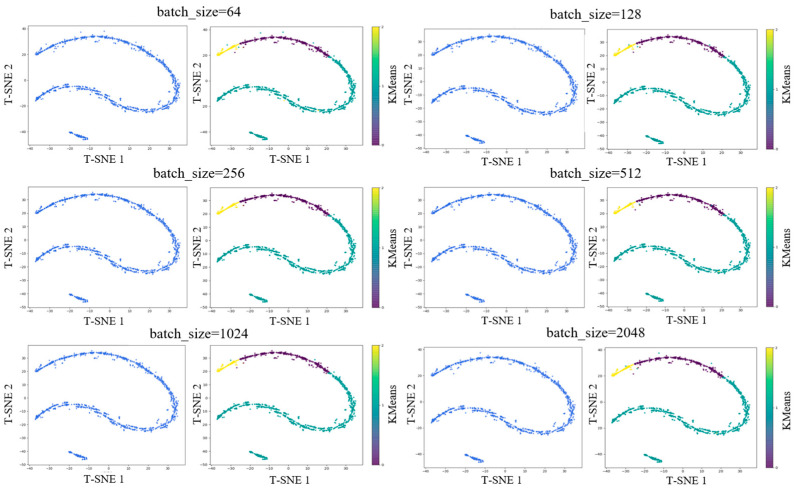
T-SNE visualization and K-means clustering results.

To further verify the effectiveness of the extracted high-dimensional features and the reliability of the batch size selection, we perform a t-SNE dimensionality reduction combined with KMeans unsupervised clustering for visual validation. The result is shown in Figure 9.

For each batch size, the evaluation includes two visualizations: a t-SNE feature distribution plot (solid color) and a t-SNE plot with KMeans clustering labels (three-color map). From the t-SNE visualization of high-dimensional features, it is evident that after reducing the learned preference features to two dimensions, a distinct banded structure emerges, forming three clearly separated strip-like clusters. This indicates that the driver preference feature extractor has learned highly discriminative representations in the high-dimensional feature space.

Moreover, previous studies on driving styles or preferences often classify drivers into three categories: aggressive, normal, and cautious. The three-cluster pattern observed in the t-SNE plot aligns well with this traditional classification scheme, suggesting that the learned features naturally capture these behavioral distinctions.

To further validate the consistency between the learned representations and clustering results, we applied unsupervised KMeans clustering directly to the high-dimensional features and assigned a cluster label to each vector. We then visualized the clustering results using t-SNE. Based on the visual structure in the t-SNE plots and the conventional three-category preference model, we set n_clusters = 3 for KMeans. The results are shown in the right side of Figure 9, where the three colors (representing KMeans cluster labels) correspond neatly to the three band-like regions in the t-SNE space. The separation is distinct with minimal overlap, indicating that the high-dimensional features can be reliably clustered into three categories. This also confirms that the observed structure is inherent in the feature space rather than an artifact of t-SNE or KMeans, further validating that the learned features align with traditional driver preference classifications. Across different batch sizes, batch_size = 256 consistently yields the best clustering effect.

It is also worth noting that while the t-SNE plots across various batch sizes remain largely similar, minor differences may appear in the positions of a few points or in classification boundaries. This variation is expected, as the goal of contrastive learning is to train the model to differentiate between different samples, and as long as training is properly conducted, the resulting feature space will converge to a consistent and meaningful structure. This confirms that the trajectory data inherently contains information reflecting three distinct driver preferences, and that the proposed driver preference feature extractor is stable and reliable.

**Figure 10:**
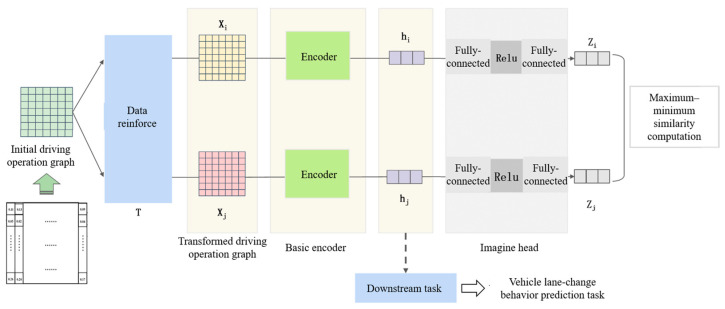
Fine tuning of the contrastive learning-based driver preference feature extraction model.

Once the SimCLR-based extractor completes training, the next step is to transfer the learned high-dimensional features to downstream tasks—this process is known as fine tuning. In this study, we retain the feature extractor (excluding the projection head) and use the extracted high-dimensional driver preference features as inputs to the downstream lane change behavior prediction task, as illustrated in Figure 10.

### 4.3. Results Analysis of the Dual-Branch Vehicle Lane Change Behavior Prediction Model

In the problem of vehicle lane change behavior prediction, Precision, Recall, and F1-score are commonly used evaluation metrics.

Precision is defined as the proportion of true positive samples among all samples predicted as positive by the model, reflecting the accuracy of the model’s positive predictions. The formula is as follows:

(12) $$Precision=\frac{TP}{TP+FP}$$

Here, TP (true positive) is the number of true positive samples, i.e., the samples correctly predicted as positive by the model; FP (false positive) is the number of false positive samples, i.e., the samples incorrectly predicted as positive by the model.

Recall is defined as the proportion of samples correctly predicted as positive among all actual positive samples. It reflects the model’s ability to capture positive samples and is calculated as follows:

(13) $$Recall=\frac{TP}{TP+FN}$$

Here, FN (false egative) is the number of false negatives, i.e., positive samples that the model incorrectly predicts as negative.

The F1-score is the harmonic mean of Precision and Recall, used to balance the trade-off between them. Its formula is as follows:

(14) $$F1=2\times \frac{Precision\times Recall}{Precision+Recall}=\frac{2\times TP}{2\times TP+FP+FN}$$

This study selected 17,590 vehicle trajectory sequences from the HighD dataset, including 8787 straight-driving sequences, 3830 left-turn sequences, and 4973 right-turn sequences. For each vehicle, 50 frames (T = 2 s) of data were selected. The training and testing datasets were split in an 8:2 ratio. The experimental environment is summarized in Table 1.

**Figure 11:**
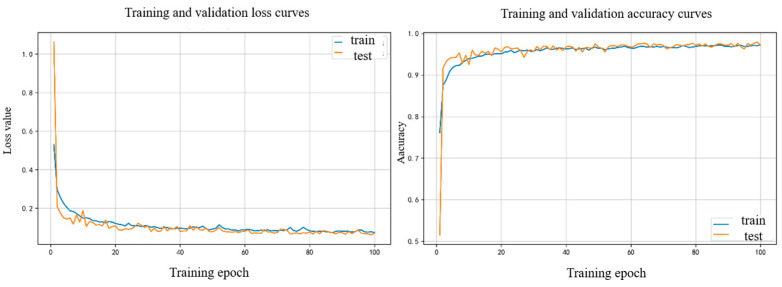
Loss and accuracy curves of the OURS model.

The model training status is shown in Figure 11 below.

For baseline selection, we chose commonly used models for vehicle lane change behavior prediction, including Transformer, LSTM, and CNN models [37]. The model results are shown in Table 2.

The behavior column in the table includes left lane change (LLC), right lane change (RLC), lane keep (LK), and the macro-average (Macro Avg).

From the overall model performance perspective, the proposed model in this paper achieves the best average results across all tasks, with an average precision of 0.9793, recall of 0.9680, and F1 score of 0.9733. The Transformer model performs second best, with an average precision of 0.9714, recall of 0.9624, and F1 score of 0.9664. The LSTM model ranks third, with an average precision of 0.9483, recall of 0.9331, and F1 score of 0.9401. The CNN model performs the worst, with an average precision of 0.9162, recall of 0.9120, and F1 score of 0.9141.

Looking at specific performance metrics for LLC (left lane change), the proposed model achieves the highest F1 score of 0.9708, precision of 0.9874, and recall of 0.9547, demonstrating the most balanced performance. The Transformer model achieves the highest precision at 0.9968 but has a lower recall of 0.9272, which reduces its F1 score to 0.9608. The LSTM model has a precision of 0.9663, recall of 0.9518, and F1 score of 0.9590, slightly lower than the previous two. The CNN model shows the weakest performance with a precision of 0.9299, recall of 0.9065, and F1 score of 0.9181.

For RLC (right lane change), the proposed model again outperforms others with the highest precision, F1 score, and recall at 0.9931, 0.9585, and 0.9755, respectively. The Transformer model’s recall is slightly lower than ours at 0.9843, with precision at 0.9479 and F1 score at 0.9658. The LSTM model’s recall is 0.9627, but precision is only 0.9270, resulting in an F1 score of 0.9445. The CNN model lags behind with a precision of 0.9092, recall of 0.9236, and F1 score of 0.9164.

Regarding LK (lane keep), the Transformer model achieves a relatively high F1 score of 0.9726 and the highest recall at 0.9757, indicating strong performance. The proposed model obtains a precision of 0.9574, recall of 0.9907, and F1 score of 0.9738. The LSTM model has a precision of 0.9516 but a lower recall of 0.8848, resulting in an F1 score of 0.9170. The CNN model performs the poorest in all three metrics with precision at 0.9096, recall at 0.9060, and F1 score at 0.9078.

In addition, we introduce the performance of the OURS model compared to a lane change prediction model without fused driver preference features. The result is shown at Table 3. Overall, the fusion model has achieved further improvements in the average performance across all metrics, with an average precision of 0.9793, a recall of 0.9680, and an F1-score of 0.9733. The improvement is most notable in terms of precision. In terms of specific performance, the fusion model has achieved significant increases in precision for RLC (right lane change) and in recall for LK (lane keep), while performing at a roughly comparable level to the baseline in other metrics.

**Figure 12:**
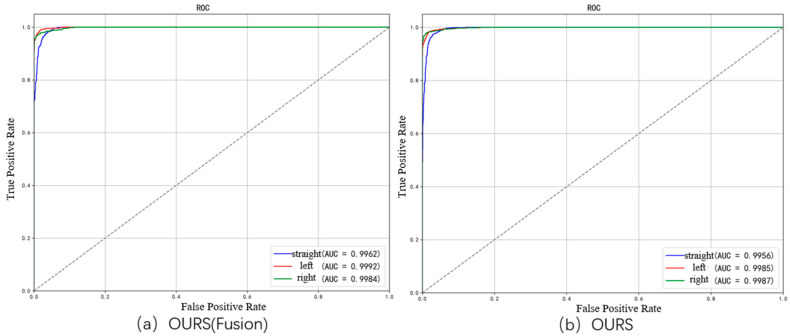
ROC curve comparison between baseline model and OURS model (with driver-preference fusion) for lane change behavior prediction.

Also, we further visualize the performance of the OURS model compared to a lane change prediction model without fused driver preference features using ROC curves. The horizontal axis represents the false positive rate (FPR), i.e., the proportion of negative samples incorrectly predicted as positive; the vertical axis represents the true positive rate (TPR), i.e., the proportion of positive samples correctly predicted. The closer the ROC curve is to the top-left corner, the better the model performance. The ROC curves are shown in Figure 12. The fusion model achieves AUC values of 0.9962, 0.9992, and 0.9984 for straight driving, left turn, and right turn, respectively. There is a significant improvement in AUC values for straight driving and left turn. This is also evident from the ROC curves, where the areas under the red curve (fusion model) are noticeably larger than the blue curve (baseline model). In summary, the fusion model improves across all metrics and achieves especially better results in left turn prediction.

## 5. Conclusions

This paper first constructs a driver preference feature extractor based on self-supervised learning. Existing lane change behavior prediction models primarily rely on explicit features, often neglecting implicit factors such as driver preferences. To address this, a contrastive learning approach within the SimCLR framework is employed to extract high-quality representations of driving preferences from DOPs. Through experiments with varying batch sizes, the optimal batch size is determined to be 256. Subsequently, using transfer learning and fine-tuning techniques, the implicit driving preference features are integrated into conventional data-driven lane change behavior prediction models via feature-level fusion. Experimental results demonstrate that the proposed fusion model achieves higher accuracy and robustness across various lane change behaviors, offering a novel approach to behavior prediction in autonomous driving.

Future research will explore the applicability of the model in complex traffic scenarios (such as dense traffic flow and sudden obstacles) and analyze failure cases under environmental disturbances like extreme weather. It is advisable to consider introducing reinforcement learning to handle dynamic vehicle–vehicle interactions, combine multi-sensor fusion to enhance robustness, and expand the training with lane-changing failure samples to improve the comprehensiveness of predictions.

## Figures and Tables

**Table 1:** Experimental environment.

Types	Configuration
Operating System	Windows 10
CPU	13th Gen Intel(R) Core (TM) i5-13600KF 3.50 GHz (Santa Clara, CA, USA)
RAM	32 GB
GPU	NVIDIA GeForce RTX 4070 (Santa Clara, CA, USA)
PyTorch and CUDA	1.13.1 + V11.6.112

**Table 2:** Comparison of experimental performance results.

Model	Behavior	T = 2 s (50 Frames)		
		Precision	Recall	F1-Score
OURS	LLC	0.9874	0.9547	0.9708
	RLC	0.9931	0.9585	0.9755
	LK	0.9574	0.9907	0.9738
	Macro Avg	0.9793	0.9680	0.9733
Transformer	LLC	0.9968	0.9272	0.9608
	RLC	0.9479	0.9843	0.9658
	LK	0.9695	0.9757	0.9726
	Macro Avg	0.9714	0.9624	0.9664
LSTM	LLC	0.9663	0.9518	0.9590
	RLC	0.9270	0.9627	0.9445
	LK	0.9516	0.8848	0.9170
	Macro Avg	0.9483	0.9331	0.9401
CNN	LLC	0.9299	0.9065	0.9181
	RLC	0.9092	0.9236	0.9164
	LK	0.9096	0.9060	0.9078
	Macro Avg	0.9162	0.9120	0.9141

**Table 3:** The comparative experiment between OURS model with fused driver preference features and model without it.

Model	Behavior	T = 2 s (50 Frames)		
		Precision	Recall	F1-Score
OURS (FUSION)	LLC	0.9874	0.9547	0.9708
	RLC	0.9931	0.9585	0.9755
	LK	0.9574	0.9907	0.9738
	Macro Avg	0.9793	0.9680	0.9733
OURS	LLC	0.9936	0.9538	0.9733
	RLC	0.9568	0.9848	0.9706
	LK	0.9721	0.9557	0.9638
	Macro Avg	0.9742	0.9648	0.9692

## Data Availability

The data and code are available from the corresponding author.

## Abbreviations

The following abbreviations are used in this manuscript:

SVM	Support Vector Machines
HMM	Hidden Markov Models
GA	Genetic Algorithm
SimCLR	Simple Contrastive Learning Framework
LLC	Left Lane Change
RLC	Right Lane Change
TP	True Positive
FP	False Positive
FN	False Negative
LSTM	Long Short-Term Memory
